# Temporal development of the oral microbiome and prediction of early childhood caries

**DOI:** 10.1038/s41598-019-56233-0

**Published:** 2019-12-24

**Authors:** S. G. Dashper, H. L. Mitchell, K.-A. Lê Cao, L. Carpenter, M. G. Gussy, H. Calache, S. L. Gladman, D. M. Bulach, B. Hoffmann, D. V. Catmull, S. Pruilh, S. Johnson, L. Gibbs, E. Amezdroz, U. Bhatnagar, T. Seemann, G. Mnatzaganian, D. J. Manton, E. C. Reynolds

**Affiliations:** 1 Oral Health Cooperative Research Centre, Melbourne Dental School, University of Melbourne, Carlton, Vic Australia; 2 ACRF Blood Cancer Therapeutics Centre, Central Clinical School, Monash University, Melbourne, Vic Australia; 3 Melbourne Integrative Genomics, School of Mathematics and Statistics, The University of Melbourne, Melbourne, Vic Australia; 4 Jack Brockhoff Child Health & Wellbeing Program, Melbourne School of Population & Global Health, University of Melbourne, Carlton, Vic Australia; 5 Department of Dentistry and Oral Health, La Trobe Rural Health School, La Trobe University, Bendigo, Vic Australia; 6 School of Health and Social Development, Deakin University, Burwood, Vic Australia; 7 Doherty Applied Microbial Genomics, Department of Microbiology and Immunology, University of Melbourne, Doherty Institute for Infection and Immunity, Melbourne, Vic Australia; 8 Department of Mathematical and Modeling Engineering, Institut National des Sciences Appliquées de Toulouse, Toulouse, France; 9 Sydney Dental Hospital, New South Wales, Australia

**Keywords:** Microbiome, Dental caries

## Abstract

Human microbiomes are predicted to assemble in a reproducible and ordered manner yet there is limited knowledge on the development of the complex bacterial communities that constitute the oral microbiome. The oral microbiome plays major roles in many oral diseases including early childhood caries (ECC), which afflicts up to 70% of children in some countries. Saliva contains oral bacteria that are indicative of the whole oral microbiome and may have the ability to reflect the dysbiosis in supragingival plaque communities that initiates the clinical manifestations of ECC. The aim of this study was to determine the assembly of the oral microbiome during the first four years of life and compare it with the clinical development of ECC. The oral microbiomes of 134 children enrolled in a birth cohort study were determined at six ages between two months and four years-of-age and their mother’s oral microbiome was determined at a single time point. We identified and quantified 356 operational taxonomic units (OTUs) of bacteria in saliva by sequencing the V4 region of the bacterial 16S RNA genes. Bacterial alpha diversity increased from a mean of 31 OTUs in the saliva of infants at 1.9 months-of-age to 84 OTUs at 39 months-of-age. The oral microbiome showed a distinct shift in composition as the children matured. The microbiome data were compared with the clinical development of ECC in the cohort at 39, 48, and 60 months-of-age as determined by ICDAS-II assessment. *Streptococcus mutans* was the most discriminatory oral bacterial species between health and current disease, with an increased abundance in disease. Overall our study demonstrates an ordered temporal development of the oral microbiome, describes a limited core oral microbiome and indicates that saliva testing of infants may help predict ECC risk.

## Introduction

Early Childhood Caries (ECC) is a complex, multifaceted disease involving interactions between the oral microbiota, host susceptibility at the tooth, mouth and person level, and environmental factors, especially behaviours relating to early feeding practices, ingestion of free sugars and oral hygiene^1–3^. ECC is defined as the presence of one or more decayed (non-cavitated or cavitated lesions), missing (due to caries), or filled tooth surfaces (dmfs) in any primary tooth in a child under the age of six years^4^. Australian children at four years of age have an average decayed, missing, and filled teeth (dmft) score of 1.94^5^. However, this score masks the true pattern and burden of disease as 60% of those examined were caries free. Additionally, the burden of disease associated with ECC is disproportionately borne by children from vulnerable and disadvantaged families^6^.

Although largely preventable ECC can be difficult to diagnose in its early stages. Cavitation is a late stage of the disease process that initially manifests as a change in the species composition of the supragingival plaque microbiota from the commensal plaque biofilm community to a dysbiotic community that is dominated by acidogenic and aciduric species^7,8^. Although *S. mutans* and to a lesser degree *S. sobrinus* have historically been linked to dental caries initiation recent microbiome-based studies have implicated a much wider range of other acidogenic and aciduric bacterial species, including other streptococci, *Actinomyces* spp. and bifidobacteria such as *Scardovia wiggsiae* with disease^9–11^. The abundance of a number of these species in supragingival plaque has been associated with caries activity in cross-sectional studies^12^. Detection of the change in composition of the supragingival plaque microbiota is therefore a method to detect ECC-risk prior to clinical manifestation of caries and offers the opportunity to treat caries at its earliest stages preventing irreversible cavitation.

The bacterial aetiology of ECC is further complicated by the fact that bacteria are colonising the child’s oral cavity at a time when teeth are erupting and are vulnerable to demineralization^13^. We currently have limited information on the timing of the colonisation of human oral cavity by bacteria and the development of the oral microbiome in infants^14^. Saliva is an easily obtained bodily fluid that contains a significant number of detectable oral bacterial species that have the potential to act as biomarkers for the bacterial dysbiosis in supragingival plaque that leads to clinical caries manifestation^13^. The bacterial composition of saliva, whilst over representing those bacteria present on the mucosal surfaces of the oral cavity, especially the tongue and buccal epithelium, has been routinely used to describe the oral microbiome^14,15^. By better understanding how the oral microbiome develops and behaves over time in infants in health and disease we may be able to identify children at highest risk of disease and develop interventions at critical time-points to more effectively prevent disease development and progression.

In this study we used a longitudinal cohort study model to characterise the oral microbiomes of 134 children at six time-points from two months-of-age to four years-of-age, along with the microbiomes of their mothers. The onset and progression of ECC in these children was determined up to five years-of-age. The oral microbiome was then compared with clinical outcomes.

## Materials and Methods

### VicGen cohort study

The VicGen longitudinal cohort study of infants/children was established in 2008 to follow the natural history and examine the causative factors of Early Childhood Caries (ECC)^16,17^. Participants were recruited through their mother from Maternal and Child Health Centres in six local government areas in Victoria, Australia. Clinical oral examinations, questionnaires and collection of saliva from the children were conducted at seven mean ages (± standard deviation) - 1.9 ± 0.8, 7.7 ± 1.3, 13.2 ± 1.2, 19.7 ± 2.0, 39.0 ± 3.2, 48.6 ± 1.6 and 60 ± 1.8 months^16,17^.

Ethics approval to conduct this study was provided by the University of Melbourne Human Research Ethics Committee (HREC 0722543 and HREC 1137124) and the study was approved by the Victorian Department of Education and Early Childhood Development (Ref: 2008/202). All research described in this manuscript was performed in accordance with relevant guidelines and regulations, and informed consent was obtained from all participants or their parent or legal guardian.

In the current nested study 134 child/mother dyads from the VicGen cohort had their saliva samples sequenced for microbiomic determination. They were selected on the basis of disease development and included a suitable number of healthy controls. Descriptive statistics were used to compare the full cohort of 259 child/mother dyads who completed the larger VicGen study with those 134 in this analysis. The 134 child/mother dyads were similar on all parameters except the larger VicGen group had a lower representation of metropolitan dwellers and as a consequence fewer lived in fluoridated areas at the beginning of the cohort study (Supplementary Table 1).

### Clinical assessment

Modified International Caries Detection and Assessment System (ICDAS II) criteria were used for caries assessment. ICDAS II was developed for clinical use in caries management and epidemiology, as the measure correlates the clinical appearance of a lesion with its histological depth or severity. ICDAS II is validated for use with children, comparable to standard criteria in epidemiological surveys^18^, and has acceptable validity and reproducibility for all codes when used with primary molar teeth^19^. The need to collect data in the field rather than a dental clinic setting precluded the use of compressed air, therefore Code 1 lesions could not be recorded unless they were located in pits or fissures. The caries assessment was conducted by three dental practitioners who had been trained, calibrated and were familiar with the screening methodology before clinical data collection^17^. Restorations were given an ICDAS II score of 5, with a total of nine children at the sixth visit and 20 children at the seventh visit having at least one dental restoration. Teeth that were reported to be extracted due to caries were given an ICDAS II score of 6. One child from the sixth visit and one from the seventh visit each had an extraction due to dental caries.

### Saliva sampling

Unstimulated saliva, up to 5 mL, was collected from infants using a pipette and from children by passive drooling into a sterile tube. Maternal saliva was collected at the same time as their infant’s second clinical assessment at 7.7 months-of-age. The samples were rapidly frozen at −20 °C, stored and periodically transported to the Melbourne Dental School where they were stored at −80 °C in secure freezers until further analysis.

### 16S rRNA gene sequencing analysis of saliva samples

In total saliva samples from 134 children and their mothers were subjected to microbiomic analyses. The children’s saliva was analysed at the first six mean ages - 1.9, 7.7, 13.2, 19.7, 39.0 and 48.6 months-of-age. Total DNA was extracted from saliva samples for each time-point collected. Targeted amplification of the V4 region of the 16S rRNA phylogenetic marker gene was performed using PCR with custom barcoded primers essentially as described previously^20^.

### DNA extraction

Methods for DNA extraction from saliva were based on the NIH Human Microbiome Project Manual of Procedures (www.ncbi.nlm.nih.gov). Total DNA was extracted from saliva using PowerLyzer® PowerSoil® DNA Isolation Kits (Qiagen). The manufacturer’s “Vacuum Protocol” instructions were followed with minor modifications as follows: For a given volume of saliva, the amount of total DNA that could be extracted was much higher for adults than for children. Therefore, for DNA extraction up to 400 μL of saliva from children and 200 μL of saliva from adults was added to the bead tubes. Bead Solution was then added to a final volume of 750 μL, as well as 4 μL of 100 mM RNase A (Sigma Aldrich) to eliminate RNA. Samples were lysed using a Precellys 24 Homogeniser (Bertin Technologies), at speed 5000 for 45 s. Detergent-containing Solution-C1 was added post-homogenisation to avoid excessive foaming and mixed by inversion. Samples were then incubated for 5 min at room temperature, to allow time for RNA digestion, before performing the inhibitor removal, binding and washing steps as specified in the Mo Bio Laboratories protocol. DNA was eluted using 40 μL of 2 mM Tris buffer at pH 8, pre-heated to 70 °C. Purified DNA was stored at −80 °C.

### PCR amplification and sequencing

The Ion Amplicon Library Preparation Fusion Method (Thermo Fisher Scientific), was adapted for amplification of the V4 region of the 16S rRNA gene from oral bacteria. Experimental controls consistently showed that the Platinum^®^ PCR SuperMix High Fidelity specified in the protocol, failed to amplify *Actinomyces naeslundii* DNA, so Q5^®^ Hot Start High-Fidelity DNA Polymerase (Genesearch) was substituted. PCRs were performed using a 50 μL reaction volume containing 6 ng of total DNA as template (consisting of ~3 ng bacterial DNA), 1X Q5 reaction buffer, 0.3 μM PAGE-purified custom barcoded primers (Thermo Fisher Scientific), 0.2 mM dNTPs (Thermo Fisher Scientific), and 1 unit of Q5 Hot Start High-Fidelity DNA polymerase. Reactions were held at 98 °C for 3 min to denature the DNA, followed by 17 cycles of amplification at 98  °C for 10 s, 48 °C for 10 s, and 72 °C for 15 s. The sequences of the 62 barcoded forward-primers and the single reverse-primer used in this study are shown in the Table of Primers (Supplementary Table 2). Amplicon libraries were purified using Agencourt® AMPure® XP Reagent (Beckman Coulter) according to the instructions provided in the Ion Amplicon Library Preparation Fusion Method protocol. Purified barcoded amplicon libraries were quantified using a LabChip GX Touch 24 nucleic acids separation system using the DNA high sensitivity kits (Perkin Elmer). Emulsion PCR of pooled amplicon libraries was performed using an Ion OneTouch™ 2 System with 400 bp protocols and multiplex sequencing was performed on an Ion Torrent Personal Genome Machine, utilising Hi-Q chemistry with 525 reagent flows per run (Thermo Fisher Scientific).

### Bioinformatics and statistical analyses

Torrent Suite Software was used to demultiplex samples and trim sequencing adapters and barcodes. The Galaxy/GVL 4.1.0 workbench and FROGS^21^ was used for filtering and clustering of sequences, essentially as described previously^20^. Primer sequences were trimmed and sequences were removed that did not contain both forward and reverse primers, were less than 220 bp in length or greater than 350 bp, contained ambiguities, contained homopolymers with size >7 nt, or contained two poor qualities scores (<10) that were <= 10 nt apart. Clustering was performed using the Swarm method^22^, chimeric DNA sequences were detected and removed as were any clusters containing only a single sequence. After trimming and filtering a total of 31,714,787 amplicon sequences remained with an average read length of 255 nt.

Statistical analyses were carried out using R CRAN version 3.4.3 and specific R packages^23^. The Phyloseq package^24^ was used to compare community structure, and DESeq. 2 was used for differential abundance analysis. The HOMD database was used to assign cluster taxonomy^25^. The multivariate longitudinal analyses were conducted as follows: OTU count table were transformed as relative proportions using Total Sum Scaling, followed by Centered Log Ratio transformation^26^. Principal Component Analysis was conducted using the mixOmics R package^27^. Because of uneven sampling on each individual infant, time trajectories for each OTU and each infant were modelled using smoothing splines with smoothing parameter 0.4. This process enabled us to interpolate matching time points across each infant. For bacterial biomarker identification, we applied sparse Partial Least Square Discriminant Analysis (sPLS-DA)^28^ where we considered specific time points of interest (e.g 39 and 48.6 months-of-age) to discriminate ECC as defined as one or more ICDAS II score of 2 or above versus healthy.

To determine if the oral microbiome composition changed before the onset of cavitation the cohort was divided into four groups based on the time of the first clinical detection of cavitation, as determined by one or more ICDAS II scores of 3 or above. The four groups were composed of children who had cavitation at 39 months-of-age, comprising 12 individuals; 33 individuals at 48.6 months-of-age; and 20 individuals who had developed disease at 60 months-of-age; and a stay healthy group who had no clinically detectable disease in the first 60 months of life, comprising 69 individuals. The oral microbiome composition was then compared with disease status at later time-points.

## Results

The oral microbiomes of 134 children were characterised at six time-points from two months up to four years-of-age. Of a possible 804 samples, 762 provided usable sequence data. The oral microbiomes of 132 of the 134 mothers were determined at a single time point. The sequenced saliva samples generated 32 million 16S DNA sequences, on average ~35,000 16S DNA sequences were identified per saliva sample with a total of 356 Operational Taxonomic Units (OTUs) identified.

### Temporal development of the oral microbiome

**Figure 1:**
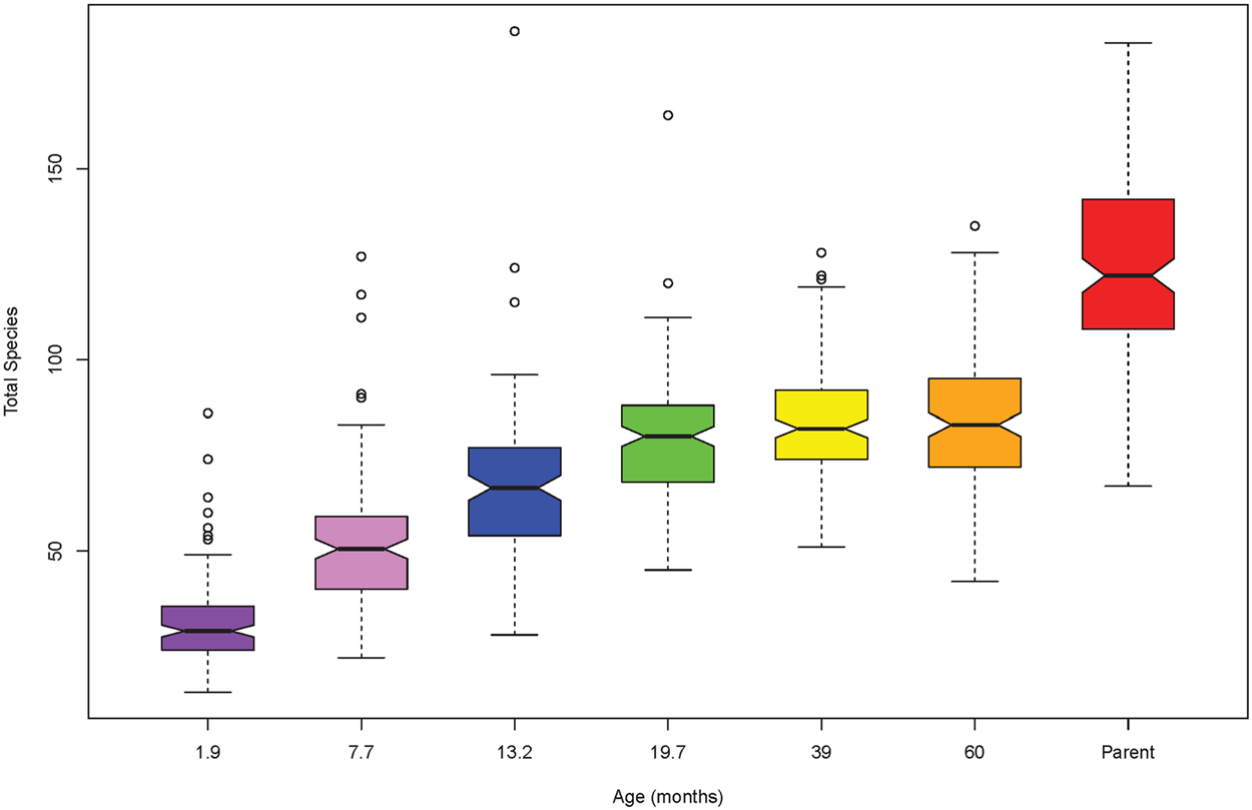
Notch plot of the bacterial α-diversity of saliva from the 134 children and their mothers examined in this study. The figure shows oral bacterial community development in children as the increase in taxa number over six time-points, from 1.9 to 48.6 months-of-age. The number of bacterial taxa in the saliva of mothers taken when their children were 7.7 months-of-age is shown for comparison. The box represents the interquartile (50% of data), the horizontal line in the box represents the median, the “notch” represents the 95% confidence interval of the median and the “whiskers” represent the maximum and minimum values.

**Figure 2:**
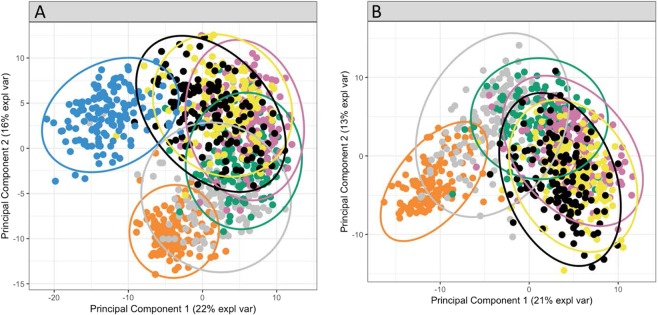
Principal component analysis of the oral microbiome from 1.9 months to 48.6 months-of-age showing the temporal development of the bacterial community. (**A**) Inclusion of the mothers’ oral microbiome (blue) into the data, showing the relationship of the children’s microbiome to that of their mothers’. (**B**) Without the mothers, a longitudinal trend is still observed. The microbiome of each child is shown at ages 1.9 (orange), 7.7 (grey), 13.2 (green), 19.7 (pink), 39 (yellow) and 48.6 (black) months. Confidence ellipse plots for each mean age group are represented at a 95% level.

**Figure 3:**
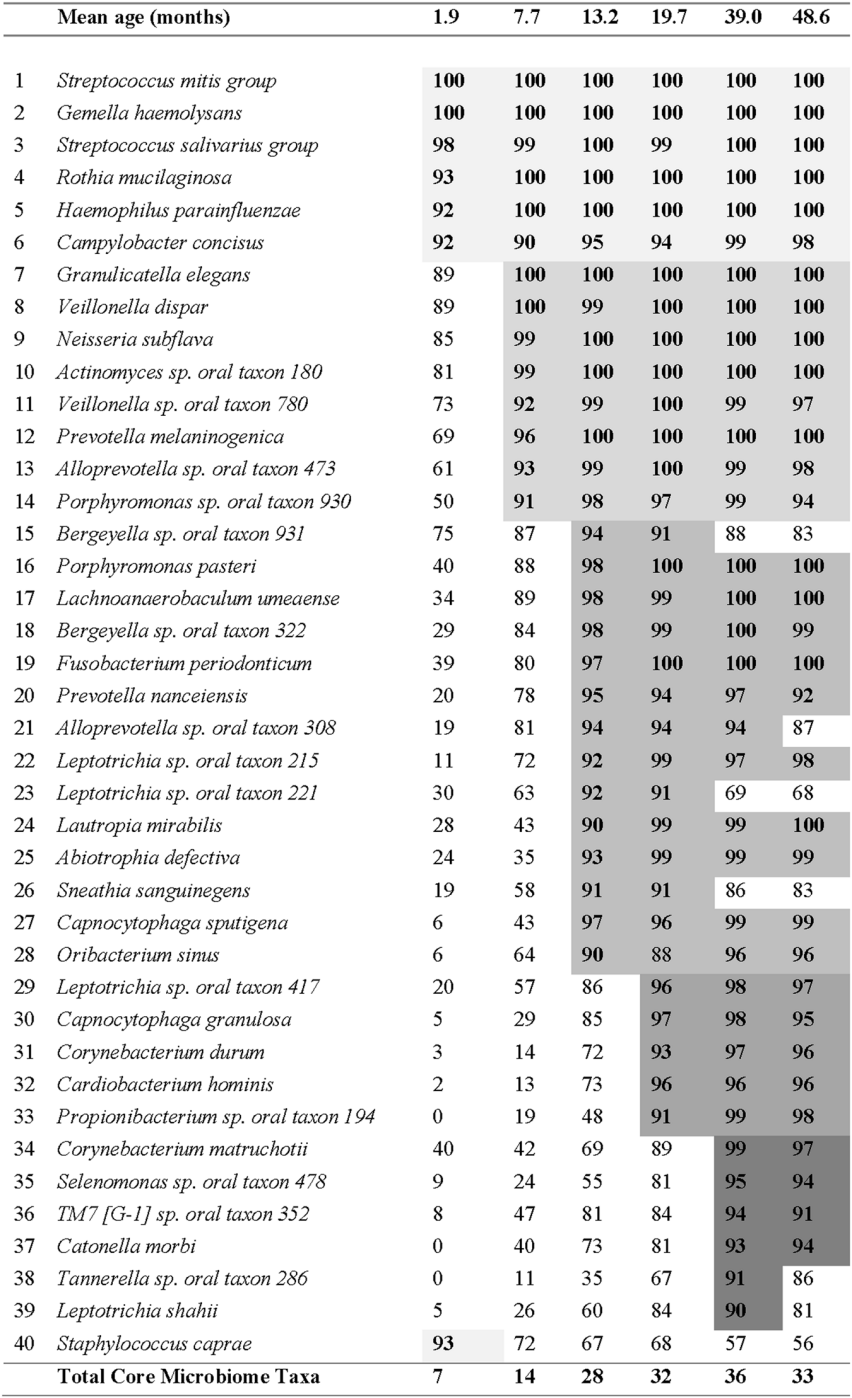
Temporal development of the core oral microbiome. Values represent the percentage of the cohort with detectable levels of each taxa at the six mean ages tested. Taxa were considered to be part of the core microbiome if they were present in the saliva of more than 90% of the children in the cohort at one or more time-points. Bold font within the Figure shows those values above 90%. Taxa are arranged from early to late colonisers and the shading clusters taxa based on when they first became part of the core microbiome. *Staphylococcus caprae* is placed at the end of the Figure due to its anomalous colonization pattern. The final row shows the total number of taxa in the children’s core microbiome at that time point.

Over time there was an increase in the mean number of bacterial OTUs in the saliva of infants. Children at 1.9 months-of-age had a low bacterial diversity in their saliva with an average of 31 ± 11 OTUs. The bacterial diversity increased over time to reach an average of 84 ± 16 OTUs at 39 months-of-age then remained stable until 48.6 months-of-age (Fig. 1). The mothers of these children had an average of 124 ± 25 OTUs in saliva. Principal component analysis of the temporal development of the oral microbiome showed a distinct shift in the composition of the microbiome over time (Fig. 2a). To aid in our understanding of how the oral microbiome develops we defined a “core oral microbiome” as containing all taxa that had a 90% or higher prevalence at one or more time-points. At 1.9 months-of-age only seven bacterial OTUs, *Streptococcus mitis* group (100%), *Gemella haemolysans* (100%), *Streptococcus salivarius* group (98%)*, Rothia mucilaginosa* (93%), *Staphylococcus caprae* (93%), *Haemophilus parainfluenzae* (92%) and *Campylobacter concisus* (92%) were found in above 90% of the child saliva samples (Fig. 3). At 7.7 months-of-age the number of species detected in the saliva of 90% or more of the cohort had increased to 14. This included six of the seven previous species with only *Staphylococcus caprae* decreasing to below 90% prevalence. At 13.2 months-of-age the core microbiome of saliva had increased to 28 species and by 19.7 months-of-age it was 32 species (Fig. 3). The total number of species in the core microbiome then remained relatively stable until 48.6 months-of-age, although the composition varied. The mothers had a distinct oral microbiome from the children (Fig. 2b) with a core microbiome of 54 species, of which 32 of the 40 species listed in Fig. 3 were present. Notable additions to the core microbiome of the adults compared with the children included five species of *Prevotella* (*P. denticola, P. salivae, P. pallens, P. oris* and *P. nigrescens*), *S. mutans* and *T. socranskii*. Interestingly a number of species including *Porphyromonas* sp. oral taxon 930 (64%), *Bergeyella* sp. oral taxon 931 (50%) and *S. sanguinegens* (40%) all decreased markedly in prevalence in adults compared with children.

**Figure 4:**
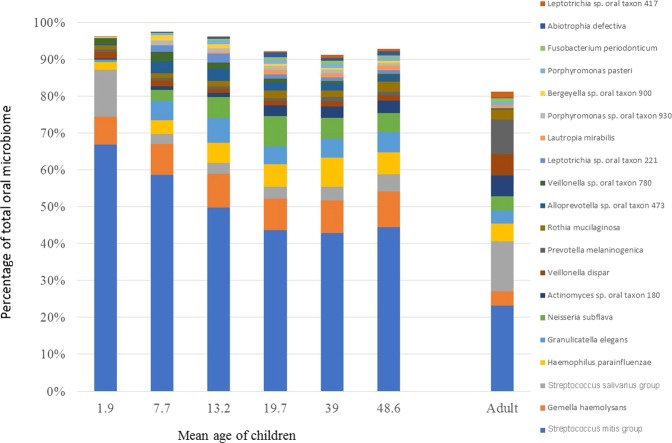
Change in the bacterial composition of children’s saliva from 1.9 months to 48.6 months-of-age based on the twenty most abundant taxa. The bacterial composition of the mothers’ saliva when the children were 7.7 months-of-age is shown for comparison.

The five most abundant OTUs, *Streptococcus mitis* group, *G. haemolysans*, *S. salivarius* group, *H. parainfluenzae*, and *Granulicatella elegans*, composed on average 90% of the total bacteria found in saliva at 1.9 months-of-age. This total proportion declined to 70% by four years-of-age driven mainly by declines in two of the OTUs the *Streptococcus mitis* group and the *S. salivarius* group (Fig. 4). At all time-points the *Streptococcus mitis* group was by far the most abundant OTU in saliva. However, the abundance of this taxon decreased substantially over the first 19.7 months of life, as the species richness of the saliva increased, and then remained relatively stable until 48.6 months-of-age. At this age the *Streptococcus mitis* group was still twice as abundant in children’s saliva compared with their mothers (Fig. 4).

*G. haemolysans* was the second most abundant bacterial species across the first four years of life. There was a relatively small increase in abundance of this species between two months and four years-of-age. Interestingly the adult saliva on average contained fewer than half the *G. haemolysans* of the children (Fig. 4). *H. parainfluenzae* showed a steady increase in abundance over the first three years of life before dropping slightly at four years-of-age to a level similar to that seen in adults. *G. elegans* was largely absent from the saliva of infants at 1.9 months-of-age but was relatively abundant by 8 months-of-age and did not change significantly in abundance over the next 40 months of life. The *S. salivarius* group was a major component of the adults’ saliva and had a similar abundance in infants at two months-of-age. Its abundance declined markedly after two months-of-age and then only increased slightly over time to four years-of-age (Fig. 4).

### Disease development in the cohort

All infants examined in this study had a healthy dentition up to a mean age of 19.7 months. At the following clinical assessment at 39 months-of-age, 12 children had cavitation, as defined by having at least one ICDAS II score of 3 or higher. By a mean of 48.6 months-of-age a total of 45 children had clinically detectable cavitation, with 33 of those children developing cavities between 39 and 48.6 months-of-age. By a mean of five years-of-age a total of 65 of the 259 children had cavities with 20 children developing enamel cavities between 48.6 and 60 months-of-age. Using a more sensitive measure of an ICDAS II score of 2, which is a distinct visual change in enamel, as a determination of clinically detectable ECC, 106 of the 259 (41%) children had disease at five years-of-age.

As would be expected the severity of ECC varied across those 65 children with disease. To quantify this variability an ECC severity score was developed by multiplying the number of teeth affected in a child by their ICDAS II score and then summing them. Children who first had ECC detected at 39 months-of-age had a mean ECC severity score of 20.5 ± 11.4 with a median of 17.0 at 60 months-of-age. Children who first had ECC detected at 48.6 months-of-age, had a mean ECC severity score of 17.3 ± 9.0 with a median of 15.0 at 60 months-of-age. This indicates that similar disease outcomes at 60 months-of-age were obtained for children developing ECC between 39 and 48.6 months-of-age. Children who first had ECC detected at 60 months-of-age had a lower mean ECC severity score of 11.6 ± 5.8 with a median of 10.0. At the severe end of the disease spectrum by 60 months-of-age 11 children had ICDAS II scores of 6 (Extensive distinct cavity with visible dentin). One child had ten teeth with detectable ECC, five of these teeth had an ICDAS II score of 5 (Distinct cavity with visible dentin) and one had a score of 6 (Table 1). Of those children with ECC the 10% with the most severe disease had an ECC severity score of 25 or above and the top 20% had an ECC severity score of 23 or above (Table 1).

**Table 1:** Severity score of ECC at 60 months-of-age.

ID	Total No. Teeth with ICDAS II ≥2	Severity Score at 60 months-of-age*
241	9	48
641	11	46
8	8	33
802	9	31
126	8	28
486	7	28
925	5	25
896	6	25
302	7	25
226	7	25
613	6	23
660	6	23
507	5	22

The severity score was determined by multiplying the number of teeth affected by the ICDAS II score of that tooth and summing the results. The Table shows the scores for the 13 individuals with the most advanced disease in this study.

### Oral microbiome and ECC

**Figure 5:**
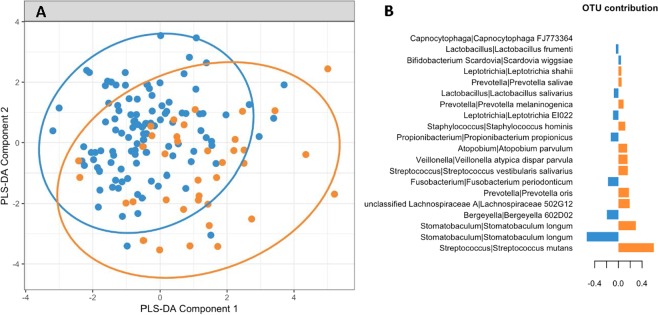
Discriminant Analysis of oral microbiome taxa at a mean age of 39 months associated with health (caries free) or disease (ECC as defined by the presence of one or more ICDAS II score of two or above). (**A**) sPLS-DA plot showing some discrimination of the sample groups, 95% confidence ellipse plots are represented. (**B**) Most important taxa selected from sPLS-DA and associated with caries free or ECC. The x-axis represents the importance of each OTU in sPLS-DA, the y-axis the OTUs with their Genus | Species taxonomy classification. Colours indicate the sample group where the OTU’s median was the largest. Blue = health; Orange = disease.

**Figure 6:**
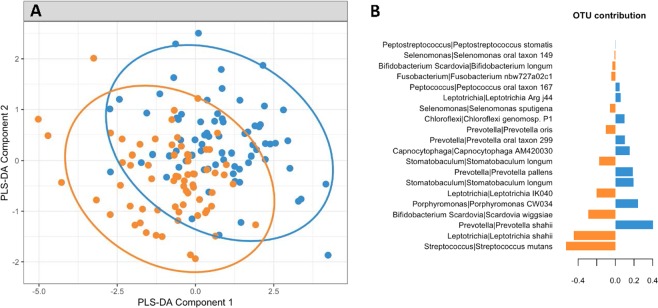
Discriminant Analysis of oral microbiome taxa at a mean age of 48.6 months associated with health (caries free) or disease (ECC as defined by the presence of one or more ICDAS II score of two or above). (**A**) sPLS-DA plot showing some discrimination of the sample groups, 95% confidence ellipse plots are represented. (**B**) Most important taxa selected from sPLS-DA and associated with caries free or ECC. The x-axis represents the importance of each OTU in sPLS-DA, the y-axis the OTUs with their Genus | Species taxonomy classification. Colours indicate the sample group where the OTU’s median was the largest. Blue = health; Orange = disease.

The sPLS-DA analysis was conducted to identify key taxa discriminating the oral microbiome of children who were healthy from those who had clinically detectable disease at 39 or 48.6 months-of-age as defined as one or more ICDAS II score of 2 or above. Overlap was observed between the two groups (Figs. 5a and 6a), but the analyses consistently identified *S. mutans* as the most discriminatory taxa associated with disease. At 48.6 months-of-age *Leptotrichia shahii*, *Scardovia wiggsiae* and *Leptotrichia* IK040 were also associated with disease (Figs. 5a and 6a). There was a less consistent association of particular taxa with health at these two ages with *Fusobacterium periodonticum*, *Stomatobaculum longum* and *Bergeyella* 602D02 being associated with health at 39 months-of-age and *Prevotella shahii*, *Prevotella pallens*, *Stomatobaculum longum*, *Porphyromonas* CW034 and *Capnocytophaga* AM20030 being associated with health at 48.6 months-of-age (Figs. 5b and 6b).

**Figure 7:**
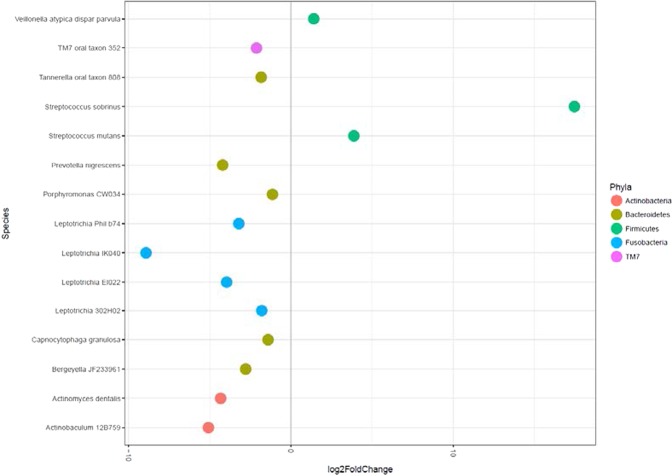
Differences in bacterial taxa abundances in saliva at 39 months-of-age in children who developed disease at 48.6 months-of-age compared with those that remained healthy.

There was little difference in the oral microbiomes at 1.9, 7.7 and 19.7 months-of-age of those children who developed disease at the later time points compared with those that remained healthy (data not shown). However, at 39 months-of-age there were differences in the abundances of particular taxa in the oral microbiome of children who developed disease compared with those who remained healthy at 48.6 months-of-age (Fig. 7). In particular *S. mutans*, *S. sobrinus* and *V. parvula* levels were all elevated in children who developed disease. Conversely *Prevotella nigrescens*, three species of *Leptotricia* and *Actinobaculum* 12B759 levels were decreased in children who developed disease.

### *S. mutans* and disease

As our data indicated that *S. mutans* was the most discriminatory taxa for the prediction of present and future clinical disease we focussed on this species. Only 24% of infants had detectable levels of *S. mutans* in their saliva at 1.9 months-of-age. Interestingly, this prevalence decreased to 17% at 7.7 months-of-age before increasing to 25% at 13.2 months-of-age, 42% at 19.7 months-of-age and 58% at 39 months-of-age. The prevalence of S. mutans then decreased slightly to 51% at 48.6 months-of-age. Nine children had no detectable *S. mutans* at any time-point up to and including the 48.6 month sample and eight of these children remained healthy with a single child developing detectable disease at 60 months-of-age.

**Figure 8:**
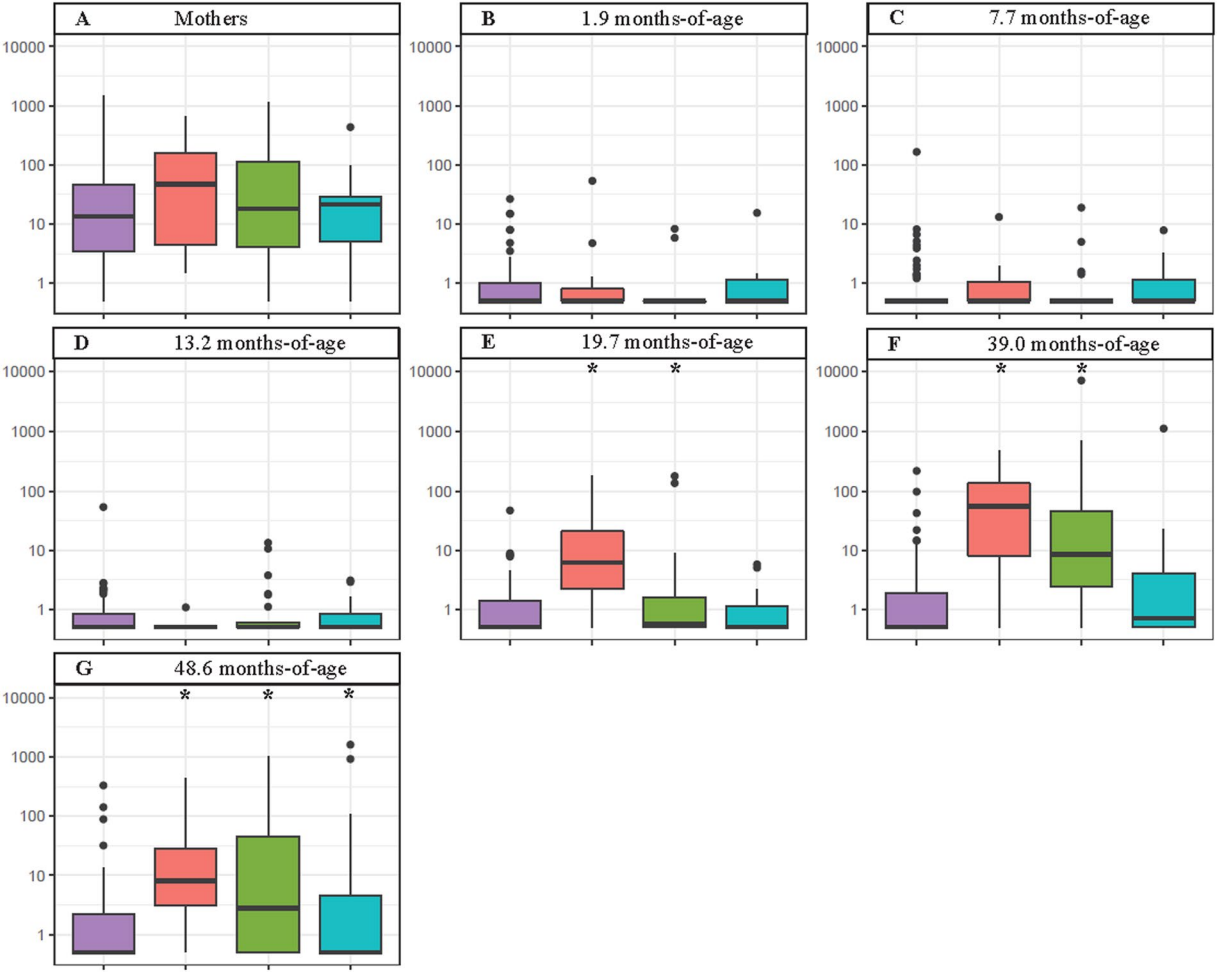
Abundance of *S. mutans* in the saliva of mothers and children at specified time points who remained healthy for the entire 60 months (purple, n = 69), developed clinically detectable disease at 39 months (red, n = 12), 48.6 months (green, n = 33) or 60 months (blue, n = 20) of age. The box plots show the median (line within the box), first and third quartiles (box), non-outlier range (whiskers), and outliers (dots). Asterisks denote those values significantly different to the remain healthy control group.

The abundance of *S. mutans* in saliva of adults at a single time-point and children at the six time-points between 1.9 months and 48.6 months-of-age was compared with the time caries was first clinically detected. As shown above ECC was first clinically detected in this cohort at 39 months-of-age. There was no significant difference in the abundance of *S. mutans* in the saliva of the mothers whose children remained healthy and those who developed clinical signs of disease at 39, 48.6 or 60 months-of-age (Fig. 8A). Similarly, *S. mutans* levels in the saliva of children at 1.9, 7.7 and 13.2 months-of-age were not significantly different in children who remained healthy compared with children who developed clinical ECC during the study (Fig. 8B–D). In contrast at 19.7 months-of-age salivary levels of *S. mutans* were significantly elevated in children who developed ECC at 39 (p = 0.017) and 48.6 (p = 0.015) months-of-age, but not children who developed disease at 60 months-of-age, compared with the stay healthy group. (Fig. 8E). At 39 months-of-age salivary levels of *S. mutans* were significantly elevated in children who first had ECC clinically detected at 39 (p = 0.000) and 48.6 (p = 0.000) months-of-age, but not those children who were first diagnosed at 60 months-of-age (p = 0.073), relative to the stay healthy group (Fig. 8F). At 48.6 months-of-age *S. mutans* levels were significantly elevated in the saliva of children who were first clinically diagnosed with caries at 48.6 (p = 0.000) and 60 (p = 0.009) months-of-age relative to the stay healthy group (Fig. 8G). At 48.6 months-of-age salivary *S. mutans* in children who were clinically determined to have ECC at 39 months-of-age was still elevated.

## Discussion

There is currently only very limited information available regarding the development of the oral microbiome in the crucial first years of life^14,29^. This is the first study to characterise the oral microbiomes of over 100 children at six time points from near birth to four years-of-age. There was a rapid colonisation of the oral cavity by a limited number of early colonising bacterial taxa over the first two years of life, demonstrating that this is a crucial age for the ordered development of the oral microbiome. The timing of colonisation and bacterial community development may be affected by a range of environmental factors including the microbiome of their mother, diet, health and antibiotic intake, and may have implications for the normal development of the oral microbiota and disease risk^29^.

Defining a core oral microbiome as being made up of species that were detected in at least 90% of the children in the cohort, regardless of the abundance of the bacterium, enabled the colonisation pattern of the oral cavity to be determined. The core oral microbiome increased five-fold between approximately two and 39 months-of-age with the initial core microbiome mainly composed of species that adhere to and colonise mucosal epithelial surfaces such as *R. mucilaginosa*, *G. haemolysans* and the *S. salivarius* and *S. mitis* groups. This is to be expected given the absence of teeth in the oral cavity of two-month-old infants. The emergence of hard non-shedding tooth surfaces and dietary changes creating new niches are likely to be responsible for the rapid expansion of the core saliva microbiome. The development of the bacterial biofilm communities that comprise plaque has been shown to proceed in an ordered manner with particular taxa fulfilling specific ecological roles^30^. Colonisation times may therefore affect normal plaque development, and possibly susceptibility to disease.

The *S. mitis* group and *G. haemolysans* were the two most abundant taxa in saliva in the first four years of life and had colonised all children by two months-of-age, demonstrating the early colonisation of the oral cavity by these species prior to tooth eruption. The *Streptococcus mitis* group are widely regarded as commensal species that are associated with oral health^31^. This group of species has a high degree of similarity at the genetic and physiological level^32^ and contains species including *S. mitis*, *S. oralis*, *S. pneumoniae, S. peroris* and *S. dentisani* that are inconsistently classified and difficult to differentiate using the V4 region of the 16s RNA gene^33^. The use of the V4 region in this study is therefore likely to lead to an underestimation of the total biodiversity within the oral microbiome. The predominance of the *S. mitis* group reduced over time as more bacterial species colonised the oral cavity. At four years-of-age both species were twice as abundant in the saliva of children compared with adults. This is consistent with the study of Dzidic *et al*.^29^ who found that *Streptococcus* spp. comprised approximately 80% of the saliva microbiome at three months-of-age declining to around 60% by 7 years-of-age and *Gemella* spp. increased from approximately 6% to 8% over the same time. Sulyanto *et al*.^14^ also found that the *S. mitis* group along with *G. haemolysans, R. mucilaginosa* and the *S. salivarius* group were the most abundant taxa in toddlers and infants up to one year of age.

In the majority of cases the prevalence of core microbiome taxa increased with age. Unusually the prevalence of *S. caprae* decreased over time. This bacterium is regarded as a commensal of human skin and its prevalence at early time-points may reflect contamination of the saliva by contact with the infant’s or mother’s skin, alternatively this species may colonise the oral cavity early in life before being displaced. Three other species *Bergeyella* sp. oral taxon 931, *Leptotrichia* sp. oral taxon 221 and *Sneathia sanguinegens* all increased in prevalence to 13.2 months-of-age and then decreased. A few species such as *Tannerella* sp. oral taxon 286, *L. shahii* and *C. morbi* increased in prevalence relatively slowly, becoming components of the core oral microbiome at 39 months-of-age. However, colonisation of the oral cavity by new species still occurs well after four years-of-age as their mothers’ saliva contained many more species and had a distinctly different composition.

In this observed cohort at a mean age of five years 105 of the 259 children in the cohort (41%) had early clinical signs of ECC as determined by an ICDAS II score of 2 or above and 65 of the children (25%) had enamel caries with cavitation, as defined as one or more teeth with an ICDAS II score of 3 or above. This level of disease is similar to that seen in comparable populations^34^. However, few epidemiological studies have been conducted with children under five years-of-age so it is difficult to generalise amongst populations^35^. ECC is a disease that is detected at varying times and progresses at different rates in individuals. As shown in this study where twelve children (4.6%) showed the first clinical evidence of disease at the 39 month examination, 33 (12.7%) showed the first clinical evidence of disease at the 48.6 month examination and 20 (7.7%) at the five year examination. As could be expected children who first displayed the clinical signs of ECC at 39 months-of-age had more advanced or widespread disease at 60 months-of-age than children who did not display clinical disease until the later time-points as determined using a cumulative ICDAS II score.

ECC begins as a change in the bacterial species composition of the plaque biofilm at specific sites in the oral cavity prior to the subsurface demineralisation of enamel and clinical symptoms of disease. Detection of the change in composition of the supragingival plaque microbiota may therefore be a way to detect ECC prior to clinical manifestation of disease and offers the opportunity to intervene to manage the disease process at its earliest stages and prevent irreversible cavitation. The abundance of a number of bacterial species in supragingival plaque has been associated with caries activity in cross sectional studies^12^. In the present study we have shown that there was a shift in the composition of the oral microbiome of children who had clinically detectable disease compared with those who were healthy at both 39 and 48.6 months-of-age. However, the magnitude of the shift was insufficient to adequately differentiate between clinical health and disease at an individual level. The most discriminatory species identified to be associated with ECC at both ages was *S. mutans*.

A number of studies have investigated the potential for the oral microbiome to predict future disease. In a recent cohort study the abundance of *Streptococcus cristatus* in saliva was significantly higher at both 3 and 24 months-of-age in children who had caries at 9 years-of-age compared with those who were caries free^29^. Sampling children at two time-points over a three-year period Holgerson and colleagues^36^ determined that apart from higher levels of lactobacilli, the bacterial composition of saliva at 3 months-of-age was unrelated to caries development at three years-of-age. The occurrence of *Lactobacillus* spp. in our cohort was universally low with usually only 2–5 children having detectable levels at any given time-point and the detection of these species showed no correlation with disease. In a population with a high prevalence of ECC Chaffee *et al*.^37^ showed that high maternal levels of *L. casei* and *S. mutans* in saliva were predictive of increased ECC occurrence. However, in the current cohort although all mothers had detectable levels of *S. mutans* in their saliva the abundance of this species was not predictive of ECC in their children.

Most children are reported to acquire *S. mutans* soon after eruption of the primary teeth^38,39^. In an American population with very high ECC incidence, 50% of the children in the cohort had detectable *S. mutans* or *S. sobrinus* in their saliva by two years-of-age and ~79% were positive by four years-of-age^40^. In the current study *S. mutans* was detectable in approximately one quarter of the cohort at 1.9 months-of-age, well before the eruption of teeth, but this prevalence decreased at 7.7 months before returning to a quarter at 13.2 months-of-age. By four years-of-age half of all children had detectable *S. mutans* in their saliva. This pattern of colonisation may indicate early inoculation from their mother or other sources followed by a failure to establish well and proliferate in some children until later in life.

At 39 months-of-age *S. mutans*, *S. sobrinus* and *V. parvula* levels in the saliva of children who developed disease at 48.6 months-of-age were all elevated compared with those that remained healthy. Salivary *S. mutans* levels were also significantly elevated at 19.7, 39 and 48.6 months-of-age in healthy children who had clinically detectable ECC at the following examination compared with children who remained healthy. Salivary *S. mutans* levels also remained elevated during active disease. It is likely therefore that salivary levels of *S. mutans*, as well as others including *S. sobrinus* and *V. parvula*, are a useful prognostic biomarker for imminent enamel demineralisation in children from 18 months to four years-of-age. Although this association has been shown previously the majority of studies specifically targeted S. mutans using PCR or culture, were cross sectional or both^41,42^. Our study using a prospective cohort study design and a relatively unbiased, open end methodology was able to examine the associations of the majority of bacterial species in saliva with disease over time. Elevated levels of *S. mutans*, *S. sobrinus* and *V. parvula* in saliva is likely to be a surrogate marker for frequent dietary carbohydrate intake in children. This intake results in dysbiosis of the microbial community leading to increases in acidogenic and aciduric bacteria in supragingival plaque^11^.

In conclusion the oral microbiome shows an ordered ecological succession during the first four years of life and the bacterial composition of saliva and in particular the abundance of certain species may have potential as biomarkers for ECC.

## Supplementary information

Supplementary Tables
