# Supplementary material for: Temporal development of the oral microbiome and prediction of early childhood caries
Source: Sci Rep. 2019 Dec 24;9:19732. doi: 10.1038/s41598-019-56233-0 (PMC6930300; doi:10.1038/s41598-019-56233-0)
Supplement: Supplementary file 1 — Supplementary Tables [file 41598_2019_56233_MOESM1_ESM.pdf]

Supplementary Data

Temporal development of the oral microbiome and prediction of early childhood caries.

Dashper SG[1*], Mitchell HL[2], Lê Cao K-A[3], Carpenter L[4], Gussy MG[5], Calache H[6], Gladman SL[7], Bulach DM[7], Hoffmann B[1], Catmull DV[1], Pruilh S[8], Johnson S[4], Gibbs L[4], Amezdroz E[4], Bhatnagar U[9], Seemann T[7], Mnatzaganian G[5], Manton DJ[1], Reynolds EC[1].

Addresses:
1. Oral Health Cooperative Research Centre, Melbourne Dental School, University of Melbourne, Carlton, Vic, Australia.
2. ACRF Blood Cancer Therapeutics Centre, Central Clinical School, Monash University, Melbourne, Vic, Australia.
3. Melbourne Integrative Genomics, School of Mathematics and Statistics, The University of Melbourne, Vic, Australia.
4. Jack Brockhoff Child Health & Wellbeing Program, Melbourne School of Population & Global Health, University of Melbourne, Carlton, Vic, Australia.
5. Department of Dentistry and Oral Health, La Trobe Rural Health School, La Trobe University, Bendigo, Vic, Australia.
6. School of Health and Social Development, Deakin University, Burwood, Vic, Australia.
7. Doherty Applied Microbial Genomics, Department of Microbiology and Immunology, University of Melbourne, Doherty Institute for Infection and Immunity, Melbourne, Vic, Australia.
8. Department of Mathematical and Modeling Engineering, Institut National des Sciences Appliquées de Toulouse, France·
9. Sydney Dental Hospital, New South Wales, Australia.

*corresponding author: stuartgd@unimelb.edu.au

Keywords: biomarkers, prognosis, cohort study, ecological succession

Running Title: Oral microbiome of children

Supplementary Table 1. Descriptive statistics comparing children from the total cohort of 259 who completed the last clinical examination whose oral microbiome was determined with children whose oral microbiome was not determined.

	Sequenced (n=134[2])			Not sequenced (n=125[3])		
	**n**	**mean**	**(sd)**	**n**	**mean**	**(sd)**
**Child birth weight**	133	3.5	(0.5)	114	3.4	(0.5)
**Pregnancy length**	133	39.3	(1.7)	116	39.3	(1.4)
**Parent age[1]**	133	31.7	(4.9)	117	31.1	(5.0)
	**n**	**%**		**n**	**%**	
**Child birth order**						
1st child	60	44.8		60	48.0	
2nd child	44	32.8		40	32.0	
3rd child	17	12.7		13	10.4	
**Parent speaks English at home**						
Yes	107	79.8		101	80.8	
**Parent born in Australia**						
Yes	96	71.6		91	72.8	
**Parent post-school education[1]**						
None	16	11.9		16	12.8	
Certificate/apprenticeship/diploma	50	37.3		43	34.4	
Bachelor degree/postgrad degree	67	50.0		58	46.4	
**Have health care card[1]**						
No	96	71.6		91	72.8	
**Household region[1]**						
Metropolitan	80	59.7		62	49.6	
Regional	22	16.4		20	16.0	
Rural	31	23.1		35	28.0	
**Residential area fluoridated [1]**						
Yes	101	75.4		81	64.8	
No	32	23.9		36	28.8	
Missing	1	0.75		8	6.4	

[1]From baseline dataset; [2] Number of children who completed the VicGen cohort study whose oral microbiome was determined; [3] [2] Number of children who completed the VicGen cohort study whose oral microbiome was not determined.

Supplementary Table 2. Primers use for DNA Amplification.

**Primer**	**Sequence (5'-3')**
trP1_V4R	CCTCTCTATGGGCAGTCGGTGATTCGGACTACHVGGGTWTCTAA
A_BC01_V4F	CCATCTCATCCCTGCGTGTCTCCGACTCAGCTAAGGTAACGAGTGCCAGCMGCCGCGGT
A_BC02_V4F	CCATCTCATCCCTGCGTGTCTCCGACTCAGTAAGGAGAACGAGTGCCAGCMGCCGCGGT
A_BC03_V4F	CCATCTCATCCCTGCGTGTCTCCGACTCAGAAGAGGATTCGAGTGCCAGCMGCCGCGGT
A_BC04_V4F	CCATCTCATCCCTGCGTGTCTCCGACTCAGTACCAAGATCGAGTGCCAGCMGCCGCGGT
A_BC05_V4F	CCATCTCATCCCTGCGTGTCTCCGACTCAGCAGAAGGAACGAGTGCCAGCMGCCGCGGT
A_BC06_V4F	CCATCTCATCCCTGCGTGTCTCCGACTCAGCTGCAAGTTCGAGTGCCAGCMGCCGCGGT
A_BC07_V4F	CCATCTCATCCCTGCGTGTCTCCGACTCAGTTCGTGATTCGAGTGCCAGCMGCCGCGGT
A_BC08_V4F	CCATCTCATCCCTGCGTGTCTCCGACTCAGTTCCGATAACGAGTGCCAGCMGCCGCGGT
A_BC09_V4F	CCATCTCATCCCTGCGTGTCTCCGACTCAGTGAGCGGAACGAGTGCCAGCMGCCGCGGT
A_BC10_V4F	CCATCTCATCCCTGCGTGTCTCCGACTCAGCTGACCGAACGAGTGCCAGCMGCCGCGGT
A_BC11_V4F	CCATCTCATCCCTGCGTGTCTCCGACTCAGTCCTCGAATCGAGTGCCAGCMGCCGCGGT
A_BC12_V4F	CCATCTCATCCCTGCGTGTCTCCGACTCAGTAGGTGGTTCGAGTGCCAGCMGCCGCGGT
A_BC13_V4F	CCATCTCATCCCTGCGTGTCTCCGACTCAGTCTAACGGACGAGTGCCAGCMGCCGCGGT
A_BC14_V4F	CCATCTCATCCCTGCGTGTCTCCGACTCAGTTGGAGTGTCGAGTGCCAGCMGCCGCGGT
A_BC15_V4F	CCATCTCATCCCTGCGTGTCTCCGACTCAGTCTAGAGGTCGAGTGCCAGCMGCCGCGGT
A_BC16_V4F	CCATCTCATCCCTGCGTGTCTCCGACTCAGTCTGGATGACGAGTGCCAGCMGCCGCGGT
A_BC17_V4F	CCATCTCATCCCTGCGTGTCTCCGACTCAGTCTATTCGTCGAGTGCCAGCMGCCGCGGT
A_BC18_V4F	CCATCTCATCCCTGCGTGTCTCCGACTCAGAGGCAATTGCGAGTGCCAGCMGCCGCGGT
A_BC19_V4F	CCATCTCATCCCTGCGTGTCTCCGACTCAGTTAGTCGGACGAGTGCCAGCMGCCGCGGT
A_BC20_V4F	CCATCTCATCCCTGCGTGTCTCCGACTCAGCAGATCCATCGAGTGCCAGCMGCCGCGGT
A_BC21_V4F	CCATCTCATCCCTGCGTGTCTCCGACTCAGTCGCAATTACGAGTGCCAGCMGCCGCGGT
A_BC22_V4F	CCATCTCATCCCTGCGTGTCTCCGACTCAGTTCGAGACGCGAGTGCCAGCMGCCGCGGT
A_BC23_V4F	CCATCTCATCCCTGCGTGTCTCCGACTCAGTGCCACGAACGAGTGCCAGCMGCCGCGGT
A_BC24_V4F	CCATCTCATCCCTGCGTGTCTCCGACTCAGAACCTCATTCGAGTGCCAGCMGCCGCGGT
A_BC25_V4F	CCATCTCATCCCTGCGTGTCTCCGACTCAGCCTGAGATACGAGTGCCAGCMGCCGCGGT
A_BC26_V4F	CCATCTCATCCCTGCGTGTCTCCGACTCAGTTACAACCTCGAGTGCCAGCMGCCGCGGT
A_BC27_V4F	CCATCTCATCCCTGCGTGTCTCCGACTCAGAACCATCCGCGAGTGCCAGCMGCCGCGGT

A_BC28_V4F	CCATCTCATCCCTGCGTGTCTCCGACTCAGATCCGGAATCGAGTGCCAGCMGCCGCGGT
A_BC29_V4F	CCATCTCATCCCTGCGTGTCTCCGACTCAGTCGACCACTCGAGTGCCAGCMGCCGCGGT
A_BC30_V4F	CCATCTCATCCCTGCGTGTCTCCGACTCAGCGAGGTTATCGAGTGCCAGCMGCCGCGGT
A_BC31_V4F	CCATCTCATCCCTGCGTGTCTCCGACTCAGTCCAAGCTGCGAGTGCCAGCMGCCGCGGT
A_BC32_V4F	CCATCTCATCCCTGCGTGTCTCCGACTCAGTCTTACACACGAGTGCCAGCMGCCGCGGT
A_BC33_V4F	CCATCTCATCCCTGCGTGTCTCCGACTCAGTTCTCATTGAACGAGTGCCAGCMGCCGCGGT
A_BC34_V4F	CCATCTCATCCCTGCGTGTCTCCGACTCAGTCGCATCGTTCGAGTGCCAGCMGCCGCGGT
A_BC35_V4F	CCATCTCATCCCTGCGTGTCTCCGACTCAGTAAGCCATTGTCGAGTGCCAGCMGCCGCGGT
A_BC36_V4F	CCATCTCATCCCTGCGTGTCTCCGACTCAGAAGGAATCGTCGAGTGCCAGCMGCCGCGGT
A_BC37_V4F	CCATCTCATCCCTGCGTGTCTCCGACTCAGCTTGAGAATGTCGAGTGCCAGCMGCCGCGGT
A_BC38_V4F	CCATCTCATCCCTGCGTGTCTCCGACTCAGTGGAGGACGGACGAGTGCCAGCMGCCGCGGT
A_BC39_V4F	CCATCTCATCCCTGCGTGTCTCCGACTCAGTAACAATCGGCGAGTGCCAGCMGCCGCGGT
A_BC40_V4F	CCATCTCATCCCTGCGTGTCTCCGACTCAGCTGACATAATCGAGTGCCAGCMGCCGCGGT
A_BC41_V4F	CCATCTCATCCCTGCGTGTCTCCGACTCAGTTCCACTTCGCGAGTGCCAGCMGCCGCGGT
A_BC42_V4F	CCATCTCATCCCTGCGTGTCTCCGACTCAGAGCACGAATCGAGTGCCAGCMGCCGCGGT
A_BC43_V4F	CCATCTCATCCCTGCGTGTCTCCGACTCAGCTTGACACCGCGAGTGCCAGCMGCCGCGGT
A_BC44_V4F	CCATCTCATCCCTGCGTGTCTCCGACTCAGTTGGAGGCCAGCGAGTGCCAGCMGCCGCGGT
A_BC45_V4F	CCATCTCATCCCTGCGTGTCTCCGACTCAGTGGAGCTTCCTCGAGTGCCAGCMGCCGCGGT
A_BC46_V4F	CCATCTCATCCCTGCGTGTCTCCGACTCAGTCAGTCCGAACGAGTGCCAGCMGCCGCGGT
A_BC47_V4F	CCATCTCATCCCTGCGTGTCTCCGACTCAGTAAGGCAACCACGAGTGCCAGCMGCCGCGGT
A_BC48_V4F	CCATCTCATCCCTGCGTGTCTCCGACTCAGTTCTAAGAGACGAGTGCCAGCMGCCGCGGT
A_BC49_V4F	CCATCTCATCCCTGCGTGTCTCCGACTCAGTCCTAACATAACGAGTGCCAGCMGCCGCGGT
A_BC50_V4F	CCATCTCATCCCTGCGTGTCTCCGACTCAGCGGACAATGGCGAGTGCCAGCMGCCGCGGT
A_BC51_V4F	CCATCTCATCCCTGCGTGTCTCCGACTCAGTTGAGCCTATTCGAGTGCCAGCMGCCGCGGT
A_BC52_V4F	CCATCTCATCCCTGCGTGTCTCCGACTCAGCCGCATGGAACGAGTGCCAGCMGCCGCGGT
A_BC53_V4F	CCATCTCATCCCTGCGTGTCTCCGACTCAGCTGGCAATCCTCGAGTGCCAGCMGCCGCGGT
A_BC54_V4F	CCATCTCATCCCTGCGTGTCTCCGACTCAGCCGGAGAATCGCGAGTGCCAGCMGCCGCGGT
A_BC55_V4F	CCATCTCATCCCTGCGTGTCTCCGACTCAGTCCACCTCCTCGAGTGCCAGCMGCCGCGGT
A_BC56_V4F	CCATCTCATCCCTGCGTGTCTCCGACTCAGCAGCATTAATTCGAGTGCCAGCMGCCGCGGT

A_BC57_V4F	CCATCTCATCCCTGCGTGTCTCCGACTCAGTCTGGCAACGGCGAGTGCCAGCMGCCGCGGT
A_BC58_V4F	CCATCTCATCCCTGCGTGTCTCCGACTCAGTCCTAGAACACGAGTGCCAGCMGCCGCGGT
A_BC59_V4F	CCATCTCATCCCTGCGTGTCTCCGACTCAGTCCTTGATGTTCGAGTGCCAGCMGCCGCGGT
A_BC60_V4F	CCATCTCATCCCTGCGTGTCTCCGACTCAGTCTAGCTCTTCGAGTGCCAGCMGCCGCGGT
A_BC61_V4F	CCATCTCATCCCTGCGTGTCTCCGACTCAGTCACTCGGATCGAGTGCCAGCMGCCGCGGT
A_BC62_V4F	CCATCTCATCCCTGCGTGTCTCCGACTCAGTTCCTGCTTCACGAGTGCCAGCMGCCGCGGT

Supplementary Table 3. Microbial signature at mean age of 39 months. Selected OTUs are ranked from the most important to the least important in the signature. The stability of each OTU is assessed via cross-validation (1 indicates that the OTU was consistently during 5-fold cross-validation repeated 50 times). Family, Genus and Species are listed. The sample group in which the OTU is mostly abundant according to its median is indicated.

	Phyla	Class	Order	Family	Genus	Species	Importance	Stability	Contribution
Cluster_48	Firmicutes	Bacilli	Lactobacillales	Streptococcaceae	Streptococcus	Streptococcus mutans	0.60	0.952	ECC
Cluster_78	Firmicutes	Clostridia	Clostridiales	Lachnospiraceae	Stomatobaculum	Stomatobaculum longum	-0.53	0.902	Caries free
Cluster_59	Firmicutes	Clostridia	Clostridiales	Lachnospiraceae	Stomatobaculum	Stomatobaculum longum	0.30	0.52	ECC
Cluster_41	Bacteroidetes	Flavobacteria	Flavobacteriales	Flavobacteriaceae	Bergeyella	Bergeyella 602D02	-0.20	0.272	Caries free
Cluster_93	Firmicutes	Clostridia	Clostridiales	Lachnospiraceae	unclassified Lachnospiraceae A	Lachnospiraceae 502G12	0.19	0.316	ECC
Cluster_71	Bacteroidetes	Bacteroidia	Bacteroidales	Prevotellaceae	Prevotella	Prevotella oris	0.18	0.244	ECC
Cluster_21	Fusobacteria	Fusobacteria	Fusobacteriales	Fusobacteriaceae	Fusobacterium	Fusobacterium periodonticum	-0.18	0.244	Caries free
Cluster_3	Firmicutes	Bacilli	Lactobacillales	Streptococcaceae	Streptococcus	Streptococcus vestibularis	0.16	0.178	ECC

						salivarius			
Cluster_7	Firmicutes	Negativicutes	Selenomonadales	Veillonellaceae	Veillonella	Veillonella atypica dispar parvula	0.15	0.174	ECC
Cluster_44	Actinobacteria	Coriobacteridae	Coriobacteriales	Coriobacteriaceae	Atopobium	Atopobium parvulum	0.15	0.148	ECC
Cluster_32	Actinobacteria	Actinobacteridae	Actinomycetales	Propionibacteriaceae	Propionibacterium	Propionibacterium propionicus	-0.13	0.1	Caries free
Cluster_20	Firmicutes	Bacilli	Bacillales	Staphylococcaceae	Staphylococcus	Staphylococcus hominis	0.12	0.182	ECC
Cluster_111	Fusobacteria	Fusobacteria	Fusobacteriales	Fusobacteriaceae	Leptotrichia	Leptotrichia EI022	-0.09	0.078	Caries free
Cluster_12	Bacteroidetes	Bacteroidia	Bacteroidales	Prevotellaceae	Prevotella	Prevotella melaninogenica	0.09	0.056	ECC
Cluster_131	Firmicutes	Bacilli	Lactobacillales	Lactobacillaceae	Lactobacillus	Lactobacillus salivarius	-0.08	0.094	Caries free
Cluster_29	Bacteroidetes	Bacteroidia	Bacteroidales	Prevotellaceae	Prevotella	Prevotella salivae	0.06	0.038	ECC
Cluster_51	Fusobacteria	Fusobacteria	Fusobacteriales	Fusobacteriaceae	Leptotrichia	Leptotrichia shahii	0.05	0.044	ECC
Cluster_110	Actinobacteria	Actinobacteridae	Bifidobacteriales	Bifidobacteriaceae	Bifidobacterium Scardovia	Scardovia wiggsiae	0.04	0.078	Caries free

Cluster_89	Firmicutes	Bacilli	Lactobacillales	Lactobacillaceae	Lactobacillus	Lactobacillus frumenti	-0.04	0.076	Caries free
Cluster_96	Bacteroidetes	Flavobacteria	Flavobacteriales	Flavobacteriaceae	Capnocytophaga	Capnocytophaga FJ773364	0.00	0.028	Caries free

Supplementary Table 4. Microbial signature at **mean age of 48.6 months.** Selected OTUs are ranked from the most important to the least important in the signature. The stability of each OTU is assessed via cross-validation (1 indicates that the OTU was consistently during 5-fold cross-validation repeated 50 times). Family, Genus and Species are listed. The sample group in which the OTU is mostly abundant according to its median is indicated.

	Phyla	Class	Order	Family	Genus	Species	Importance	Stability	Contribution
Cluster_48	Firmicutes	Bacilli	Lactobacillales	Streptococcaceae	Streptococcus	Streptococcus mutans	-0.53	1.00	ECC
Cluster_51	Fusobacteria	Fusobacteria	Fusobacteriales	Fusobacteriaceae	Leptotrichia	Leptotrichia shahii	-0.45	0.96	ECC
Cluster_107	Bacteroidetes	Bacteroidia	Bacteroidales	Prevotellaceae	Prevotella	Prevotella shahii	0.41	0.91	Caries free
Cluster_110	Actinobacteria	Actinobacteridae	Bifidobacteriales	Bifidobacteriaceae	Bifidobacterium Scardovia	Scardovia wiggsiae	-0.29	0.64	ECC
Cluster_19	Bacteroidetes	Bacteroidia	Bacteroidales	Porphyromonadaceae	Porphyromonas	Porphyromonas CW034	0.25	0.47	Caries free
Cluster_84	Fusobacteria	Fusobacteria	Fusobacteriales	Fusobacteriaceae	Leptotrichia	Leptotrichia IK040	-0.20	0.30	ECC
Cluster_78	Firmicutes	Clostridia	Clostridiales	Lachnospiraceae	Stomatobaculum	Stomatobaculum longum	0.20	0.31	Caries free
Cluster_33	Bacteroidetes	Bacteroidia	Bacteroidales	Prevotellaceae	Prevotella	Prevotella pallens	0.19	0.31	Caries free

Cluster_59	Firmicutes	Clostridia	Clostridiales	Lachnospiraceae	Stomatobaculum	Stomatobaculum longum	-0.17	0.26	ECC
Cluster_95	Bacteroidetes	Flavobacteria	Flavobacteriales	Flavobacteriaceae	Capnocytophaga	Capnocytophaga AM420030	0.16	0.15	Caries free
Cluster_30	Bacteroidetes	Bacteroidia	Bacteroidales	Prevotellaceae	Prevotella	Prevotella oral taxon 299	0.10	0.10	Caries free
Cluster_71	Bacteroidetes	Bacteroidia	Bacteroidales	Prevotellaceae	Prevotella	Prevotella oris	-0.10	0.13	ECC
Cluster_67	Chloroflexi	Chloroflexi	Chloroflexales	Chloroflexaceae	Chloroflexi	Chloroflexi genomosp. P1	0.10	0.09	Caries free
Cluster_91	Firmicutes	Negativicutes	Selenomonadales	Veillonellaceae	Selenomonas	Selenomonas sputigena	-0.06	0.04	ECC
Cluster_25	Fusobacteria	Fusobacteria	Fusobacteriales	Fusobacteriaceae	Leptotrichia	Leptotrichia Arg j44	0.06	0.05	Caries free
Cluster_72	Firmicutes	Clostridia	Clostridiales	Peptococcaceae	Peptococcus	Peptococcus oral taxon 167	0.05	0.06	Caries free
Cluster_135	Fusobacteria	Fusobacteria	Fusobacteriales	Fusobacteriaceae	Fusobacterium	Fusobacterium nbw727a02c1	-0.04	0.00	ECC
Cluster_63	Actinobacteria	Actinobacteridae	Bifidobacteriales	Bifidobacteriaceae	Bifidobacterium Scardovia	Bifidobacterium longum	-0.03	0.01	ECC

Cluster_38	Firmicutes	Negativicutes	Selenomonadales	Veillonellaceae	Selenomonas	Selenomonas oral taxon 149	-0.01	0.03	ECC
Cluster_54	Firmicutes	Clostridia	Clostridiales	Peptostreptococcaceae	Peptostreptococcus	Peptostreptococcus stomatis	0.00	0.02	Caries free

	Phyla	Class	Order	Family	Genus	Species	Importance	Stability	Contribution
Cluster_48	Firmicutes	Bacilli	Lactobacillales	Streptococcaceae	Streptococcus	Streptococcus mutans	0.60	0.952	ECC
Cluster_78	Firmicutes	Clostridia	Clostridiales	Lachnospiraceae	Stomatobaculum	Stomatobaculum longum	-0.53	0.902	Caries free
Cluster_59	Firmicutes	Clostridia	Clostridiales	Lachnospiraceae	Stomatobaculum	Stomatobaculum longum	0.30	0.52	ECC
Cluster_41	Bacteroidetes	Flavobacteria	Flavobacteriales	Flavobacteriaceae	Bergeyella	Bergeyella 602D02	-0.20	0.272	Caries free
Cluster_93	Firmicutes	Clostridia	Clostridiales	Lachnospiraceae	unclassified Lachnospiraceae A	Lachnospiraceae 502G12	0.19	0.316	ECC
Cluster_71	Bacteroidetes	Bacteroidia	Bacteroidales	Prevotellaceae	Prevotella	Prevotella oris	0.18	0.244	ECC
Cluster_21	Fusobacteria	Fusobacteria	Fusobacteriales	Fusobacteriaceae	Fusobacterium	Fusobacterium periodonticum	-0.18	0.244	Caries free
Cluster_3	Firmicutes	Bacilli	Lactobacillales	Streptococcaceae	Streptococcus	Streptococcus vestibularis	0.16	0.178	ECC

						salivarius			
Cluster_7	Firmicutes	Negativicutes	Selenomonadales	Veillonellaceae	Veillonella	Veillonella atypica dispar parvula	0.15	0.174	ECC
Cluster_44	Actinobacteria	Coriobacteridae	Coriobacteriales	Coriobacteriaceae	Atopobium	Atopobium parvulum	0.15	0.148	ECC
Cluster_32	Actinobacteria	Actinobacteridae	Actinomycetales	Propionibacteriaceae	Propionibacterium	Propionibacterium propionicus	-0.13	0.1	Caries free
Cluster_20	Firmicutes	Bacilli	Bacillales	Staphylococcaceae	Staphylococcus	Staphylococcus hominis	0.12	0.182	ECC
Cluster_111	Fusobacteria	Fusobacteria	Fusobacteriales	Fusobacteriaceae	Leptotrichia	Leptotrichia EI022	-0.09	0.078	Caries free
Cluster_12	Bacteroidetes	Bacteroidia	Bacteroidales	Prevotellaceae	Prevotella	Prevotella melaninogenica	0.09	0.056	ECC
Cluster_131	Firmicutes	Bacilli	Lactobacillales	Lactobacillaceae	Lactobacillus	Lactobacillus salivarius	-0.08	0.094	Caries free
Cluster_29	Bacteroidetes	Bacteroidia	Bacteroidales	Prevotellaceae	Prevotella	Prevotella salivae	0.06	0.038	ECC
Cluster_51	Fusobacteria	Fusobacteria	Fusobacteriales	Fusobacteriaceae	Leptotrichia	Leptotrichia shahii	0.05	0.044	ECC
Cluster_110	Actinobacteria	Actinobacteridae	Bifidobacteriales	Bifidobacteriaceae	Bifidobacterium Scardovia	Scardovia wiggsiae	0.04	0.078	Caries free
Cluster_89	Firmicutes	Bacilli	Lactobacillales	Lactobacillaceae	Lactobacillus	Lactobacillus frumenti	-0.04	0.076	Caries free
Cluster_96	Bacteroidetes	Flavobacteria	Flavobacteriales	Flavobacteriaceae	Capnocytophaga	Capnocytophaga FJ773364	0.00	0.028	Caries free
